# Therapeutic Potential of Gnetin C in Prostate Cancer: A Pre-Clinical Study

**DOI:** 10.3390/nu12123631

**Published:** 2020-11-26

**Authors:** Ketaki Gadkari, Urvi Kolhatkar, Rutu Hemani, Gisella Campanelli, Qing Cai, Avinash Kumar, Anait S. Levenson

**Affiliations:** 1 Arnold & Marie Schwartz College of Pharmacy and Health Sciences, Long Island University, Brooklyn, NY 11201, USA; Ketaki.gadkari@my.liu.edu (K.G.); Urvi.kolhatkar@my.liu.edu (U.K.); Rutu.hemani@my.liu.edu (R.H.); Gisella.campanelli@my.liu.edu (G.C.); Qing.cai@liu.edu (Q.C.); Avinash.kumar@liu.edu (A.K.); 2 College of Veterinary Medicine, Long Island University, Brookville, NY 11548, USA

**Keywords:** Gnetin C, xenografts, therapy, MTA1, prostate cancer

## Abstract

Natural stilbenes have gained significant attention in the scientific community owing to their potential anticancer effects against prostate cancer. We recently reported that Gnetin C, a resveratrol (Res) dimer, demonstrated more potent inhibition of metastasis-associated protein 1/v-ets avian erythroblastosis virus E26 oncogene homolog 2 (MTA1/ETS2) axis in prostate cancer cell lines than other stilbenes. In this study, we investigated in vivo antitumor effects of Gnetin C in two doses (50 and 25 mg/kg, i.p.) using PC3M-Luc subcutaneous xenografts and compared these to Res and pterostilbene (Pter). We found that while vehicle-treated mice revealed rapid tumor progression, compounds-treated mice showed noticeable delay in tumor growth. Gnetin C in 50 mg/kg dose demonstrated the most potent tumor inhibitory effects. Gnetin C in 25 mg/kg dose exhibited tumor inhibitory effects comparable with Pter in 50 mg/kg dose. Consistent with the effective antitumor effects, Gnetin C-treated tumors showed reduced mitotic activity and angiogenesis and a significant increase in apoptosis compared to all the other groups. The data suggest that Gnetin C is more potent in slowing tumor progression in prostate cancer xenografts than Res or Pter. Taken together, we demonstrated, for the first time, that Gnetin C is a lead compound among stilbenes for effectively blocking prostate cancer progression in vivo.

## 1. Introduction

Epidemiological studies indicate a link between diet and incidence and mortality of certain types of cancer including prostate cancer [1,2]. According to the American Cancer Society, prostate cancer accounts for approximately 11% of newly diagnosed cancers and 5.4% of cancer-caused death in the US. Out of all the newly diagnosed prostate cancer cases, the mortality rate is 17.3%. While some diets, such as high-fat diet, red meats, and dairy products, may play a role as DNA damage-causing carcinogens in prostate cancer [3], vegetarian diet is advocated as an important source of cancer-inhibiting bioactive polyphenols [4,5]. Numerous cohort and case-control studies support a notion that consumption of certain diets is associated with a decreased risk of prostate cancer [6,7,8,9]. Recognition of the importance of dietary polyphenols in cancer development and progression has promoted research in not only cancer chemoprevention, but also in tumor recurrence risk reduction, due to the ability of polyphenols to potentiate chemo or/and radiotherapy [10,11,12]. Therefore, more often, polyphenols are considered for cancer interception and therapy, since potential structural modifications, combinations, and delivery systems are considered for increasing bioavailability and/or biological effects of these natural compounds [12,13].

A number of bioactive polyphenols have been isolated from green tea, soybeans, cruciferous vegetables, tomatoes, carrots, grapes, and other fruits and berries, which have shown significant potential to inhibit cancer progression and metastasis acting through multiple molecular mechanisms including epigenetic processes [14,15,16].

Motivated by epidemiologic data showing reduced prostate cancer risk associated with red wine consumption, which was attributed to high resveratrol (Res) content [17], stilbenoids, or stilbenes have been extensively tested in in vitro and in vivo studies showing great potential as anticancer agents in prostate cancer. Resveratrol and its analogs act on multiple targets in different signaling pathways related to inflammation, tumor cell survival and apoptosis, angiogenesis, drug resistance, invasion, and metastasis in prostate cancer [18,19,20,21,22]. Particularly, our group systematically reported on the chemopreventive and therapeutic roles of metastasis-associated protein 1 (MTA1)-mediated anticancer effects of Res, pterostilbene (Pter), and other stilbenes against prostate cancer [18,23,24,25].

Gnetin C, a Res dimer naturally found in grapes or melinjo plant, has recently been discovered to possess potent biological properties, including anti-inflammatory and anticancer properties [26], and has shown no toxicity in humans [27]. This prompted us to study its antitumor activity in prostate cancer. We recently reported that Gnetin C acts through MTA1/ETS2-mediated mechanisms in prostate cancer and shows significant MTA1-mediated inhibitory effects on cell viability, colony formation, and migration while inducing cell cycle arrest and cell death [28].

In the current study, we, for the first time, performed comparative evaluation of the in vivo efficacy of Gnetin C, Res and Pter in the treatment of an established tumor and not in cancer chemoprevention. In addition to evaluating anticancer and antimetastatic effects of Gnetin C in two aggressive prostate cancer cell lines in vitro, we utilized PC3M-Luc prostate cancer xenograft model to compare and contrast pre-clinical efficacy of Gnetin C with Res and Pter. We found that Gnetin C is more potent than Res and Pter in exerting its anticancer activity in prostate cancer both in vitro and in vivo, representing a novel and effective potential therapeutic agent in prostate cancer.

## 2. Materials and Methods

### 2.1. Materials

Gnetin C was a generous gift from Hosoda SHC Co., Ltd. (Fukui, Japan). Resveratrol was purchased from Sigma-Aldrich (St. Louis, MO, USA) and Pter was a gift from late Dr. AM Rimando. All compounds had ≥99% purity. Compounds were dissolved in pure dimethyl sulfoxide (DMSO, 0.1% final concentration) and stored in the dark at −20 °C.

### 2.2. Cell Lines

DU145 and PC3M prostate cancer cell lines were grown in RPMI 1640 media (Thermo Fisher Scientific, Waltham, MA, USA) containing 10% fetal bovine serum and maintained in an incubator at 37 °C with 5% CO_2._ For in vitro experiments involving treatment with Res, Pter and Gnetin C, cells were grown in conventional media which was replaced with phenol red-free RPMI 1640 containing 5% charcoal-stripped serum 16–18 h prior to treatment. The PC3M cells tagged with luciferase (PC3M-Luc) [29] were used to generate prostate cancer xenografts (see below). Cell lines were used for experiments in this study within 10–15 passages from thawing. Cells were authenticated using STR profiling at Research Technology Support Facility, Michigan State University and found to be mycoplasma-free (Universal Mycoplasma Detection Kit, ATCC, Manassas, VA, USA)

### 2.3. Cell Viability Assay

MTT cell viability assay was performed using DU145 and PC3M prostate cancer cells under treatment with Res, Pter and Gnetin C as described earlier [25,28,30,31]. Briefly, the cells were seeded into 96-well plates, treated with compounds for 72 h and the absorbance was measured using Synergy-4 plate reader (BioTek, Winooski, VT, USA). Values of vehicle-treated control cells were assigned as 100%, and the percentage of cell viability of compound-treated cells was calculated. IC_50_ was calculated using GraphPad Priszm v7 (GraphPad Software, La Jolla, CA, USA).

### 2.4. Cell Proliferation Assay

The cell proliferation assay was performed during 10–12 days for DU145 and PC3M cells. The 2.5 × 10^3^ cells were seeded in 35 mm culture dishes in replicates and treated with Res, Pter and Gnetin C. Cells were counted every alternate day post staining with Trypan blue. Experiments were performed three times for each cell line.

### 2.5. Flow Cytometry

Cell cycle analysis was performed by flow cytometry of propidium iodide (PI) as before [28]. Briefly, cells were plated at 1 × 10^6^/well in 60 mm dishes and treated with compounds for 24 h, after which they were fixed in ice-cold 95% ethanol, washed in PBS and stained with PI. The cell suspension was run through CytoFLEX and cell cycle distribution was calculated using Cytexpert software (Beckmann Coulter Inc., Miami, FL, USA).

### 2.6. Colony Formation Assay

Cells were seeded in 35 mm culture dishes and treated with compounds every day for approximately 14 days observation time as described previously [28,29,30]. When colonies were formed, cells were fixed and stained with 0.01% crystal violet solution. Images of colonies were taken using an Amersham Imager 600 and analyzed using ImageQuant TL software (GE Healthcare Bio-Sciences, Pittsburg, PA, USA).

### 2.7. Wound Healing Assay

Wound healing assay was performed as described previously [28,29,30,32]. Wounds were scratched through the 90% confluent cells, which were starved with 0.1% serum overnight. Images of wounds were taken using EVOS VL Core microscope (Thermo Fisher Scientific, Waltham, MA, USA) and analyzed using the ImageJ software (NIH, Bethesda, MD, USA). Values of vehicle-treated control cells at 0 h were assigned as 100%, and the percentage of wound area of compound-treated cells was calculated on that basis.

### 2.8. Western Blot Analysis

Western blots were performed as described previously [23,28,30]. Briefly, protein lysates from tumor tissues or cells treated with compounds for 24 h were prepared using RIPA buffer (Thermo Fisher Scientific, Waltham, MA, USA), protein was estimated, and samples were separated using 10–15% polyacrylamide gels. Protein was transferred onto polyvinylidene difluoride (PVDF) membranes, blocked with 5% milk/PBS/0.1% Tween, and then probed with primary antibodies listed in Supplementary Table S1. β-actin was used as a loading control. Signals were detected using enhanced chemiluminescence (ThermoFisher Scientific, Waltham, MA, USA). Band intensity was measured using Image J (NIH, Bethesda, MD, USA).

### 2.9. Human Prostate Cancer Xenografts

Animal housing, care, and experimental design were in accordance with approved protocol (# AL Gnetin C, May 2019) by the Institutional Animal Care and Use Committee (IACUC) of Long Island University (LIU). Male Foxn1^nu/nu^ mice (4–5 weeks-old) obtained from Envigo RMS (Indianapolis, IN, USA) were implanted subcutaneously (s.c.) on their flank with 10^6^ PC3M-Luc cells in 100 µL of 1:1 PBS/Matrigel (BD Biosciences, Bedford, MA, USA). Animals were randomly assigned to the following five groups: Control-vehicle (I); Res (II, 50 mg/kg); Pter (III, 50 mg/kg); Gnetin C (IV, 50 mg/kg), and Gnetin C (V, 25 mg/kg). The concentration of compounds were single 50 mg/kg bw dose for groups II–IV based on published effective dose in tumor xenografts [18,23,33,34,35]. Compounds were formulated in 10% DMSO and administered every day intraperitoneally (i.p.). For efficacy studies, compound treatment started when tumor volume was ~200 mm^3^ and mice were sacrificed at day 21 after treatment or when tumor volume reached 1500 mm^3^. Tumor volume was measured using Vernier digital caliper or by BL imaging weekly (see below). Tumor volume (V) was calculated using V (mm^3^) = (Length × Width^2^)/2 formula as before [18,36]. Body weights were measured twice weekly. Animals were monitored daily for signs of toxicity. At sacrifice, tumors were excised, measured, and divided into portions for histological and IHC analysis (placed in 10% formalin), for protein isolation, and for Ultra Performance Liquid Chromatography (UPLC) (snap frozen). Blood also was collected at sacrifice, and serum samples were prepared and stored at −80 °C.

### 2.10. Bioluminescent Imaging of Cells and Tumors

Prior to inoculation into mice, PC3M-Luc cells were checked for luciferase expression as described previously [23,29]. Briefly, cells were serially diluted in a black, clear bottom 96-well plate (Costar, Corning, NY, USA) and 15 mg/mL D-luciferin (Perkin Elmer, Hopkinton, MA, USA) was added to each well. Plates were incubated at 37 °C, 5% CO_2_ for 10 min, after which images were taking using IVIS Lumina LT III (Perkin Elmer, Hopkinton, MA, USA) (Supplementary Figure S1A). For in vivo BL imaging, mice were anesthetized with 2% isoflurane and i.p. injected with 150 mg d-luciferin/kg bw and placed inside the camera box as we have done previously [18,23,25,29,33,37]. Image analysis and bioluminescent quantification was performed using Living Image software (Perkin Elmer, Hopkinton, MA, USA). Normalization was done for all the images at the end of the experiment (Supplementary Figure S1B).

### 2.11. Immunohistochemistry

Tumors were dissected, fixed in 10% formalin and sent to Reveal Biosciences Inc. (San Diego, CA, USA) for tissue sectioning, H&E staining and preparation of slides for IHC staining. Immunochemistry was performed as described previously [33,37,38] using Vectastain ABC Elite Kit and ImmPACT DAB kit (Vector Laboratories, Burlingame, CA, USA). Antibodies used for IHC are listed in Supplementary Table S1. Images were viewed and recorded using EVOS XL Core microscope (Thermo Fisher Scientific, Waltham, MA, USA). Positively stained cells were counted in five randomly selected fields using ImageTool software (NIH, Bethesda, MD, USA), expressed as area (CD31) or percentage of total cells (ki67, MTA1, CC3) and analyzed by GraphPad Prism v7 (GraphPad Software, La Jolla, CA, USA).

### 2.12. Analysis of Gnetin C Content in Tumor Samples

Tumor tissue samples were homogenized and extracted by 1 mL acetonitrile and 200 μL of diluent (0.1 % formic acid and acetonitrile at a ratio of 7:3 respectively). The supernatant was air dried and reconstituted in 200 μL of diluent. A total of 10 μL samples were injected into the Ultra Performance Liquid Chromatography (UPLC) (Waters ACQUITY UPLC system, Milford, MA, USA). To construct calibration curves, fresh standards were prepared by spiking 200 μL of standard and 1 mL acetonitrile to control tissue. The calibration curve for Gnetin C was found to be linear within the range of 20–500 ng/mL (r^2^ = 0.998). The retention time for Gnetin C was 4.2 min. Acquired data were processed with Empower 3 software. Gnetin C concentrations were calculated by using the calibration curve (ng/mL) with further unit conversion to analyte vs. tumor tissue weight (ng/mg).

### 2.13. Statistical Analyses

All the in vitro experiments were performed at least three times and data is shown as mean ± SEM. Statistical analysis of tumor volume differences (on day 40) between multiple treatment groups and in vitro experiments involving comparison of Res and Pter with two doses of Gnetin C was conducted using two-way ANOVA to determine the significance of differences between different groups and doses. For in vitro experiments involving comparison of Gnetin C, Res and Pter in a single dose, statistical analysis was performed using one-way ANOVA. Statistical significance was set as *p* ≤ 0.05. Graphs were generated using GraphPad Prism v7 software (GraphPad Software, La Jolla, CA, USA).

## 3. Results

### 3.1. Gnetin C Exhibits Potent Cytotoxicity in Prostate Cancer Cells

**Figure 1:**
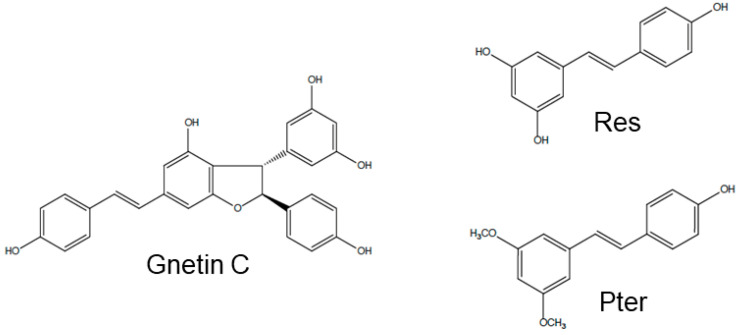
Chemical structures of Gnetin C (MW: 454.4 g/mol), Resveratrol (Res, MW: 228.3 g/mol), and Pterostilbene (Pter, MW: 256.3 g/mol).

**Figure 2:**
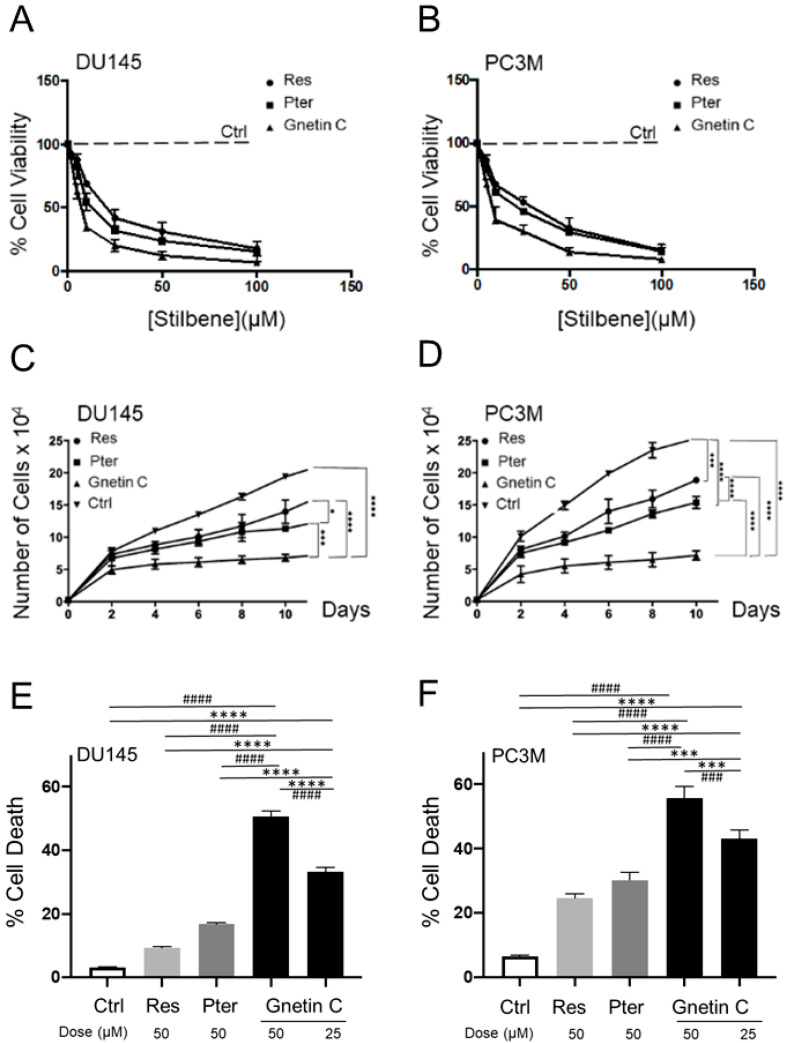
Gnetin C induces cytotoxicity in DU145 and PC3M prostate cancer cells more potently than Res and Pter. (**A**,**B**) A dose-dependent (5–100 µM) cell viability assay was performed after 72 h of treatment with compounds. Viable cells were plotted as percent of vehicle treated cells (Ctrl), which was set as 100%. (**C**,**D**) Effects of Gnetin C, Res and Pter on prostate cancer cell proliferation. Cells were plated in phenol-red-free media supplemented with 5% charcoal-stripped serum and cultured in the presence of 5 μM of compounds for 10 days. (**E**,**F**) Analysis after treatment with compounds was performed using Flow Cytometry and sub-G1 population was calculated. Data represent the mean ± SEM of three independent experiments. Data represent the mean ± SEM of three independent experiments, in which each data point was performed in triplicates. * *p* < 0.05; *** *p* < 0.001; **** *p* < 0.0001 (significance vs. Gnetin 25); ^###^ *p* < 0.001; ^####^ *p* < 0.0001 (significance vs. Gnetin 50) (two-way ANOVA).

Gnetin C, Res, and Pter belong to the stilbene class of polyphenols with ~450–230 g/mol molecular weight (MW), respectively. Resveratrol and Pter have a C6-C2-C6 basic skeleton and consist of two phenol groups linked by an ethane double bond. Pterostilbene is a naturally occurring methoxylated analog of Res, while Gnetin C is a Res dimer. Chemical structures of Gnetin C (2alpha-(4-hydroxyphenyl)-3beta-(3,5-dihydroxyphenyl)-6-[2-(4-hydroxyphenyl)ethenyl]-2,3-dihydrobenzofuran-4-ol), Res (3,4′,5-trihydroxy-*trans*-stilbene) and Pter (3,5-dimethoxy-4′-hydroxy-trans-stilbene) are shown in Figure 1. Cell viability assay showed potent dose-dependent induction of cytotoxicity by all three compounds in both cell lines. IC_50_ values for Gnetin C were 6.6 µM and 8.7 µM compared to IC_50_ for Res (21.8 µM and 24.4 µM) and Pter (14.3 µM and 19.0 µM) for DU145 and PC3M, respectively, indicating significantly more potency of Gnetin C compared to Res and Pter in both cell lines (Figure 2A,B). We then examined the effects of Gnetin C on the proliferation of DU145 and PC3M prostate cancer cells in comparison with Res and Pter. Data showed the most potent cell growth inhibitory effects of Gnetin C compared to Res and Pter in both cell lines (Figure 2C,D).

To investigate the kinetics of Gnetin C-induced cell cycle distribution in DU145 and PC3M cells, we performed cell cycle analysis using flow cytometry. Cells treated with 25 and 50 μM of Gnetin C and 50 µM of Res and Pter showed a cell population with reduced DNA content, which was detected as a sub-G1 peak in the flow cytometric histogram. The number of sub-G1 phase dead cells increased markedly in the Gnetin C-treated cells compared to Ctrl and Res- or Pter-treated cells (*p* < 0.0001) (Figure 2E,F). Altogether, Gnetin C induces more cell death in both prostate cancer cell lines.

### 3.2. Gnetin C Potently Inhibits In Vitro Metastatic Potential of Prostate Cancer Cells

**Figure 3:**
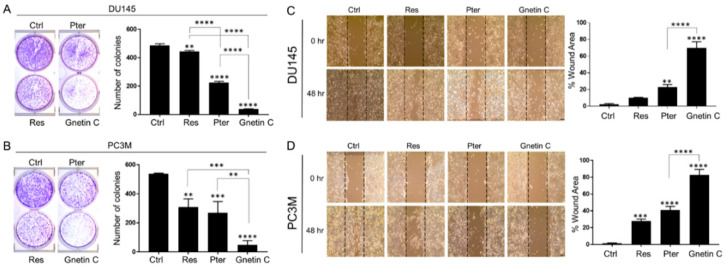
Gnetin C reduces clonogenic survival and motility in DU145 and PC3M prostate cancer cells more potently than Res and Pter. (**A**,**B**) Representative images (**left**) and quantification (**right**) of the number of colonies showing colony formation ability of prostate cancer cells after treatment with 5 μM of compounds for 14 days. Data represent the mean ± SEM of three independent experiments with duplicate wells. ** *p* < 0.01; *** *p* < 0.001; **** *p* < 0.0001 (one-way ANOVA). (**C**,**D**) Representative images (**left**) and quantification (**right**) of the migration ability of cells after treatment with 1 µM of compounds for 48 h. Values of vehicle-treated Ctrl cells at 0 h were assigned as 100%, and the percentage of wound area of compound-treated cells was calculated on that base. 10X images (scale bar, 100 µm). Data represent the mean ± SEM of six separate wound areas from three independent experiments. ** *p* < 0.01; *** *p* < 0.001; **** *p* < 0.0001 (one-way ANOVA).

To characterize the ability of Gnetin C to affect metastatic potential of cells, we performed clonogenic cell survival assay using DU145 and PC3M cells. Cells were treated with 5 μM and 10 μM of each compound for 14 days. The extent of dose-dependent inhibition of colony formation was significantly greater in Gnetin C-treated cells with major reduction in size and number of colonies compared to Res- and Pter-treated cells (Figure 3A,B).

Analysis of wound healing assay of cells cultured in the presence of 1 μM and 5 μM of Gnetin C, Res and Pter for 24 or 48 h demonstrated that all three compounds induce reduction of the wound healing compared to vehicle-treated control cells. Once again, Gnetin C exhibited the most profound effects in both cell lines and highly significant inhibition of cell migration (*p* < 0.0001) compared to Res and Pter (Figure 3C,D).

### 3.3. Gnetin C Inhibits Tumor Growth in PC3M-Luc Xenografts

**Figure 4:**
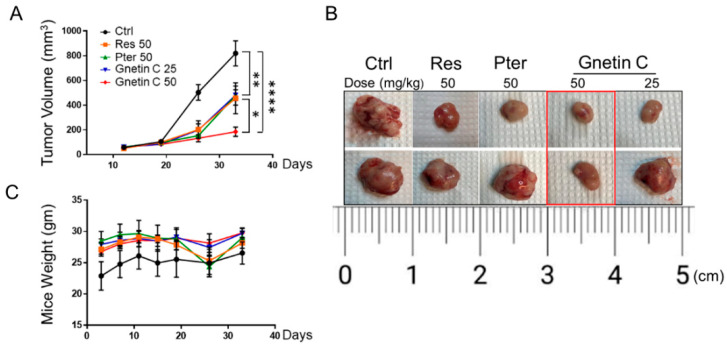
Gnetin C reduces tumor growth in PC3M-Luc s.c. xenografts. (**A**) Male nude mice were injected s.c. with 10^6^ PC3M-Luc cells. Treatment (i.p.) with compounds started at day 19 when tumor volume was ~200 mm^3^. Tumor growth was measured using a digital caliper, twice a week. Significant tumor volume reduction was detected in all compound-treated vs. Ctrl group starting at day 25 with profound effect of Gnetin C at the high dose (50 mg/kg bw) at the endpoint. The mean ± SEM are shown. * *p* < 0.05; ** *p* < 0.01; **** *p* < 0.0001 (two-way ANOVA). (**B**) Representative images of two excised tumors from each treatment group are shown: Gnetin C in 50 mg/kg bw dose had firm inhibitory effect. (**C**) Average body weights of Ctrl and compound-treated groups during the study (*n* = 7/group).

To evaluate the in vivo anticancer effects of Gnetin C, we utilized s.c. PC3M-Luc xenograft model. The animals were randomized into five groups (*n* = 7/each): vehicle-treated Ctrl; Res-treated (50 mg/kg); Pter-treated (50 mg/kg), Gnetin C (50 mg/kg), and Gnetin C (25 mg/kg). To evaluate the therapeutic potential of Gnetin C, we started i.p. treatment with compounds only after tumors reached 200 mm^3^. As shown in Figure 4A, while all three compounds inhibited tumor growth compared to vehicle-control group, Gnetin C at the same 50 mg/kg dose used for Res and Pter showed much more potent efficacy (*p* < 0.0001 vs. *p* < 0.01). Of note, at twice less 25 mg/kg dose, Gnetin C showed comparable effects with Pter, which has been shown previously to be more potent than Res in prostate cancer xenografts at the same 50 mg/kg dose [23]. At the endpoint (day 40), mice were sacrificed, and tumors were excised and photographed (Figure 4B). While we observed noticeable heterogeneity among mice in response to treatments, in agreement with our in vitro data, Gnetin C in its high 50 mg/kg bw dose consistently showed the utmost efficacy in inhibiting tumor volume. There were no significant changes in body weights or visible signs of toxicity over the course of the experiment (Figure 4C).

We also monitored tumor growth and effects of treatment by BL imaging as we have done previously in various xenografts models [18,23,29,33,37]. A validation of luciferase expression in PC3M-Luc cells was performed using bioluminescent assay in vitro (Supplementary Figure S1A) prior to transplantation. However, while we were able to monitor PC3M-Luc tumor growth in all the groups, we detected considerable intra- and inter- group variations and by the time we started treatments with compounds (day 19), luciferase signals were close to saturation in mice. Regrettably, we were not able to calculate tumor inhibitory effects of compounds by BL imaging with acceptable rigor due to highly saturated signals and inter-individual biological heterogeneity of aggressively growing PC3M-Luc tumors (Supplementary Figure S1B).

### 3.4. Gnetin C Inhibits Proliferation and Angiogenesis and Promotes Apoptosis in PC3M-Luc Xenografts

**Figure 5:**
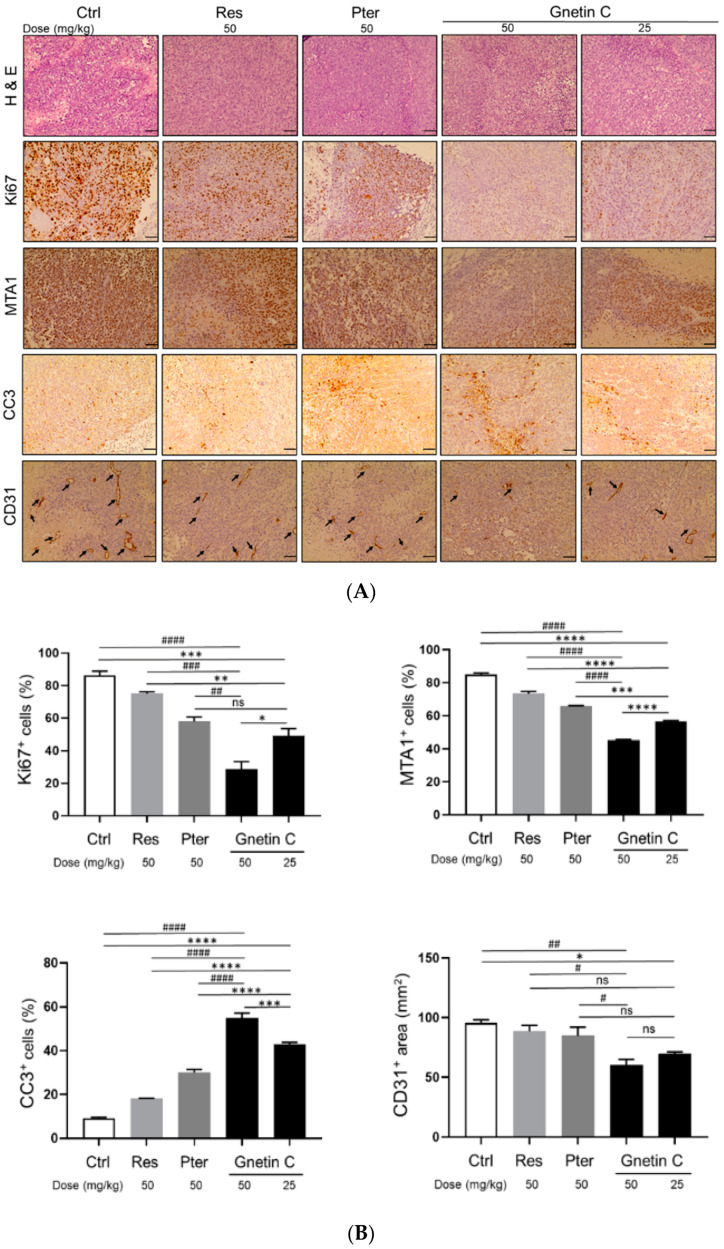
Gnetin C inhibits proliferation and angiogenesis, and induces apoptosis in PC3M-Luc s.c. xenografts. (**A**) Representative images of H&E (**top**), Ki67 (**second from top**), MTA1 (**third from top**) cleaved caspase 3 (**second from bottom**), and CD31 (**bottom**)-stained sections of the tumor tissues from mice treated with compounds. H & E, MTA1, CC3 and CD31 are 20× images (scale bar, 50 μm); Ki67 is 40× image (scale bar, 20 μm). (**B**) Quantitative analysis of immunostainings showing drastic effect of Gnetin C treatment on markers of proliferation (Ki67), cell survival (MTA1), apoptosis (CC3), and angiogenesis (CD31) in tumor tissues. Arrows indicate microvessels on the bottom panels. Values are means ± SEM of cells counted in five separate fields per sample (*n* = 7/group), and the average count is expressed as a percent. ^ns^ non-significant; * *p* < 0.05; ** *p* < 0.01; *** *p* < 0.001; **** *p* < 0.0001 (significance vs. Gnetin 25); ^#^ *p* < 0.01; ^##^ *p* < 0.01; ^###^ *p* < 0.001; ^####^ *p* < 0.0001 (significance vs. Gnetin 50) (two-way ANOVA).

The antitumorigenic effect of Gnetin C was confirmed by significant reduction of Ki67 staining and by the presence of cleaved caspase 3 (CC3)-positive cells in treated mice (Figure 5A,B). While all three compounds could decrease cell proliferation rate, notably, Gnetin C-treated tumors even in low 25 mg/kg dose showed statistically significant reduction in cell proliferation compared to effects by Res and Pter in 50 mg/kg dose. Further, consistent with our in vitro data and previous reports on stilbenes’ ability to induce apoptosis in prostate tumor tissues [23,24], treatment with the compounds resulted in significant increase in CC3-positive staining. Once again, Gnetin C in both doses exhibited statistically significant higher level of apoptosis compared to Res and Pter (Figure 5A,B). In addition, in agreement with our previous observations on the inhibitory effects of stilbenes on angiogenesis [24,25,36], treatment with the compounds led to decreased angiogenesis as evident by CD31 staining of tumor tissues. Yet again, Gnetin C inhibited angiogenesis more potently compared to Res and Pter (Figure 5A,B). Altogether, IHC analysis of tumors at sacrifice confirmed the presence of actively proliferating tumor cells only in tissues obtained from control mice while showing beneficial effects by all compounds and most potently by Gnetin C, in both doses, on cell proliferation, apoptosis and angiogenesis.

### 3.5. Gnetin C Inhibits MTA1 and MTA1-Associated Signaling

**Figure 6:**
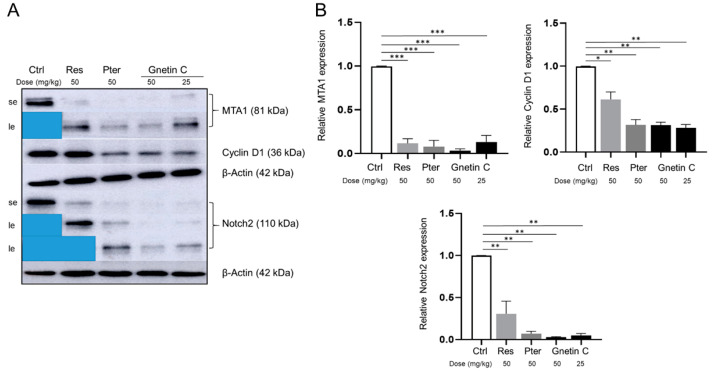
Gnetin C inhibits MTA1, Cyclin D1, and Notch 2 in PC3M-Luc s.c. xenografts. Representative immunoblot images (**A**) and densitometric analysis (**B**) of MTA1, Cyclin D1 and Notch2 levels detected in tumor tissues. Se, short exposure; le, long exposure. Values are means ± SEM of three independent experiments. β-Actin was a loading control. * *p* < 0.05; ** *p* < 0.01; *** *p* < 0.001 (two-way ANOVA).

In our previous studies, we reported on MTA1-mediated anticancer effects of Gnetin C in vitro [28]. We demonstrated that Gnetin C inhibited MTA1-mediated cell viability, clonogenic survival, migration and induced MTA1-mediated apoptosis more potently than Res and Pter in prostate cancer cells specifically acting through MTA1/ETS2 pathway [28,39]. To follow up on our in vitro observations (Supplementary Figure S2), herein, we examined the possible involvement of MTA1 and MTA1 signaling in the antitumor effects of Gnetin C in vivo. Immunohistochemical analysis of the tumor tissues showed high MTA1 expression in control mice and significant MTA1 downregulation in mice treated with all the compounds (Figure 5A,B). Notably, we were able to detect more potent and statistically significant MTA1 downregulation by Gnetin C in both doses compared to Pter, which was the lead MTA1 inhibitory stilbene in our previous studies [23,24,33]. Among the MTA1-associated genes previously identified by our group from MTA1-ChIP analysis were Cyclin D1 and Notch 2, which were shown to be responsive to Pter treatment and directly regulated by MTA1 in prostate cancer cell lines [24]. Western blot analysis of xenograft tumor tissues confirmed a strong MTA1, Cyclin D1 and Notch 2 downregulation upon treatment with compounds compared to control vehicle-treated tissues. Markedly, Gnetin C demonstrated the most potency in inhibiting these molecules (Figure 6A,B).

### 3.6. Analysis of Gnetin C Content in Tumor Tissues

We analyzed the concentration of Gnetin C in tumor samples taken at sacrifice using UPLC (Supplementary Figure S3). We can accept that Gnetin C had greater accumulation in tumor tissues than Res and Pter because we were able to detect it in both groups of mice treated with Gnetin C whereas concentrations of Res and Pter were not detectable. In the group V (Gnetin C, 25 mg/kg bw) tumors, average tissue concentration reached 0.105 ng/mg. This is ten times more than 0.014 ng/mg Res concentration previously detected by us in LNCaP xenografts after dosing by oral gavage [18].

## 4. Discussion

Numerous studies support the use of Res in cancer chemoprevention; however, due to its low bioavailability and rapid metabolism, Res did not progress into practice [40,41]. Over the past decade, various Res analogs as well as oligomers have been described and intensively studied for their more potent biological efficacy in cancer [16,24,26,39,42,43,44,45].

Resveratrol oligomers are formed by the polymerization of two or more Res units to generate dimers, trimers, tetramers, and derivatives that are more complex. Currently, more than 300 Res oligomers have been characterized [46]. In *Vitis vinifera* grapes, the most common source of Res, the concentration of Res and Res oligomers varies largely according to the grape cultivar, the geographic origin, and exposure to infections and UV light. Resveratrol oligomers have multiple beneficial biochemical and pharmacological properties, of which some are superior in stability and activity compared to Res [45]. Gnetin C is a Res dimer that is accumulated in melinjo plant (*Gnetum gnemon* L.). It has been shown that melinjo seed extract (MSE) exhibits antioxidant, antimicrobial, immunomodulatory, and anticancer effects [47,48,49,50]. Importantly, safety of MSE has been demonstrated in humans [51,52]. On the other hand, the antitumor properties of pure Gnetin C compared to its monomer Res were first demonstrated in tumor angiogenesis model [49]. ERK1/2 and AKT/mTOR mediated antileukemia effects of Gnetin C were also demonstrated in acute myeloid leukemia xenografts [53]. To determine feasibility of Gnetin C as a pharmacological agent that can be (1) combined with chemotherapeutic drugs for clinical applications, and (2) provide a backbone for new drug discovery and development requires its evolution in vivo for particular organ site cancer. To the best of our knowledge, no studies have been conducted to test the effects of pure Gnetin C in preclinical studies for prostate cancer.

We first characterized the effects of Gnetin C in prostate cancer cell lines and showed that Gnetin C more potently inhibits cell proliferation and metastatic properties of prostate cancer cells, along with inducing more degree of cell death compared to Res and Pter. In a xenograft model, we detected a decrease of tumor volume in mice treated with Res (50), Pter (50) and Gnetin C at 25 mg/kg bw dose, while a double dose of Gnetin C (50 mg/kg bw) caused a substantial reduction in tumor volume in a small cohort of mice. Hence, as expected, the tumor reduction was related to the substantial decrease in tumor cell proliferation and angiogenesis together with an increase in apoptosis, which reflected more potent activity of Gnetin C compared to Res and Pter. On a molecular level, we found superior inhibitory effect on MTA1, Cyclin D1 and Notch2 by Gnetin C compared to Res and Pter.

Further, we detected higher levels of Gnetin C in tumor tissues, which is consistent with the reported longer half-life and lower clearance of Gnetin C compared to Res and Pter [54,55].

In the current study, BL imaging did not provide additional information over volume measurements. One has to bear in mind that s.c. tumors grow unpredictably large and get signal saturation especially when aggressive cell line such as PC3M-Luc is used. The differences in tumor growth can be attributed to differences in injected Luc-tagged cells, and growth-associated structural changes such as necrosis can change the signal irrespective of treatment. Our experience emphasizes that BL imaging of s.c. tumors is a measure that is influenced by a complex mixture of parameters related to tumor microstructure and not to imaging protocol. This complexity leads to difficulties in monitoring therapeutic effects, and explains our inability to obtain any meaningful BL imaging data in the current study.

In summary, for the first time, our preclinical study using prostate cancer xenograft model and i.p. intervention of Gnetin C demonstrated successful reduction of tumor volume and inhibition of tumor progression. We found that Gnetin C has the same antitumor effects as Res and Pter in twice-lower dose. Notably, Gnetin C at the same dose as Res and Pter (50 mg/kg bw) exhibited significantly more efficacy compared to Res and Pter. However, although our study demonstrated promising preclinical antitumor effects of Gnetin C in prostate cancer, the preliminary scope of the current study is restricted to s.c. xenograft model. In general, since clinical tumors grow more slowly than preclinical xenografts, more adequate prostate cancer cells, i.e., LNCaP or DU145 should be used in the upcoming experiments. Moreover, additional pre-clinical studies including orthotopic or transgenic prostate cancer models and combination strategies with conventional /targeted anticancer drugs are warranted to substantiate these initial findings.

## 5. Conclusions

In conclusion, we suggest that Gnetin C is a potent natural product agent with vast antitumor potential in prostate cancer. In the future, human clinical trials are needed to determine whether Gnetin C can prevent prostate tumor growth in a defined subpopulation of patients diagnosed with early stage prostate cancer and whether it can be used as a therapeutic strategy, most likely in combination with approved anticancer agents.

## Supplementary Materials

The following are available online at https://www.mdpi.com/2072-6643/12/12/3631/s1, Figure S1: (A) Validation of specificity and sensitivity of Luc expression in PC3M-Luc cells in vitro. (B) Representative bioluminescent (BL) images of mice bearing PC3M-Luc tumors in each treatment groups are shown from day 19 when treatments began. Images were taken once per week by using IVIS Imaging System. Although Gnetin50 groups shows smallest image, the heterogeneity and saturated signals unable us for satisfactory quantification of differences in BL signals among the groups, Figure S2: Stilbenoid compounds inhibit MTA1 expression in prostate cancer DU145 and PC3M-Luc cells. Gnetin C consistently demonstrated more potent inhibition of MTA1 compared to Res and Pter. Protein levels were assessed by western blot. β-actin was a loading control, Figure S3: The overlaid UPLC chromatograms for A, blank tissue extraction; B, Gnetin C standard, 200 ng/mL; C, Gnetin C, 25 mg tissue sample, Figure S4: Original gels from Figure 6. Short exposure showed signals only for control tumors, in which expression of MTA1 (top gels) and Notch2 (bottom gels) were high. Masking control signals with foil and using longer exposure allowed us to detect lower expression of MTA1 and Notch2, and differences in tumors treated with compounds, Table S1: Primary antibodies used in this study.
