# Supplementary material for: Therapeutic Potential of Gnetin C in Prostate Cancer: A Pre-Clinical Study
Source: Nutrients. 2020 Nov 26;12(12):3631. doi: 10.3390/nu12123631 (PMC7760540; doi:10.3390/nu12123631)
Supplement: Supplementary file 1 [file nutrients-12-03631-s001.pdf]

Supplementary Data

K Gadkari *et al* ***Therapeutic potential of Gnetin C in prostate cancer: a pre-clinical study***

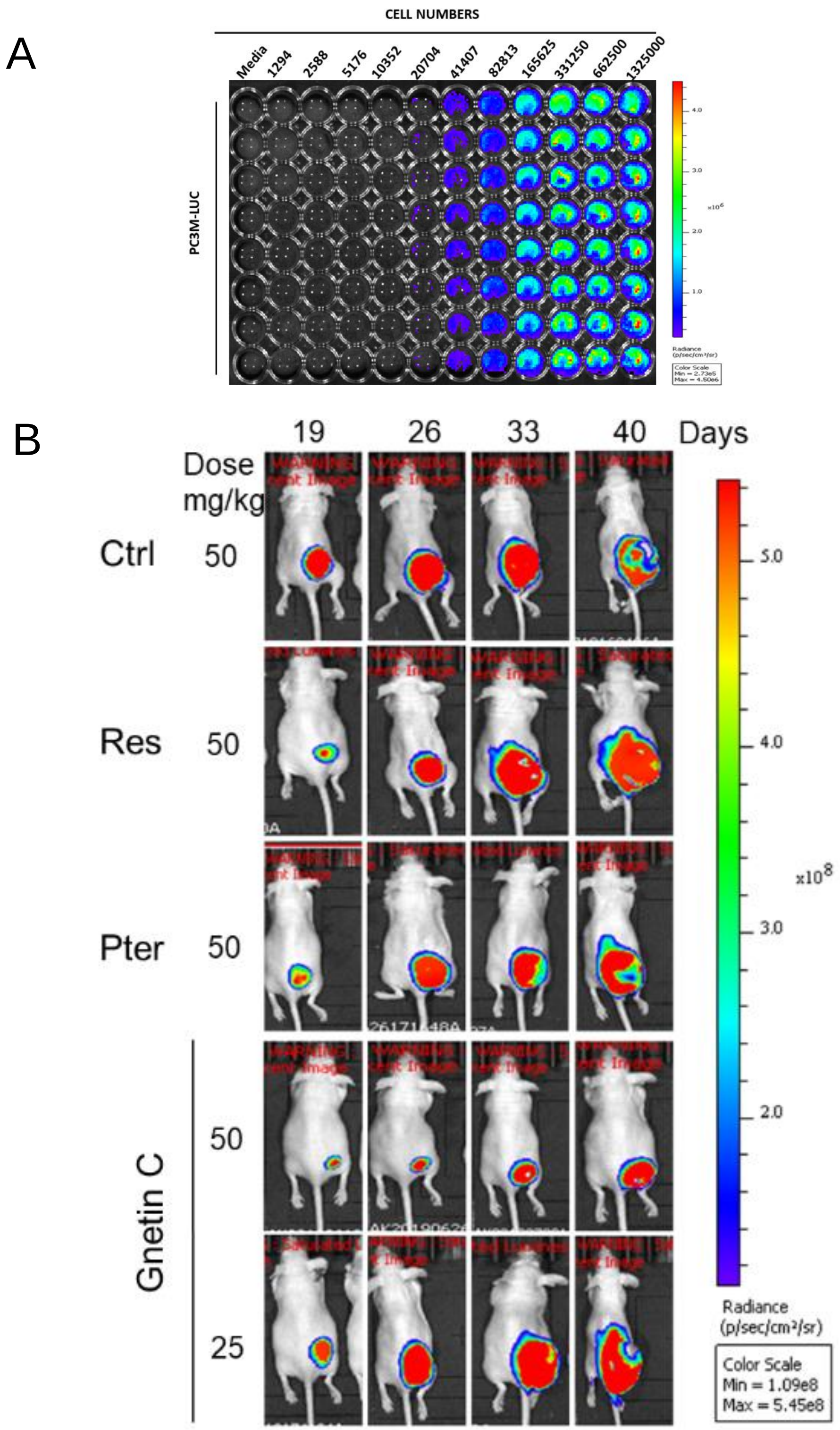

Figure S1. (**A**) Validation of specificity and sensitivity of Luc expression in PC3M-Luc cells *in vitro*. (**B**) Representative bioluminescent (BL) images of mice bearing PC3M-Luc tumors in each treatment groups are shown from day 19 when treatments began. Images were taken once per week by using IVIS Imaging System. Although Gnetin50 groups shows smallest image, the heterogeneity and saturated signals unable us for satisfactory quantification of differences in BL signals among the groups.

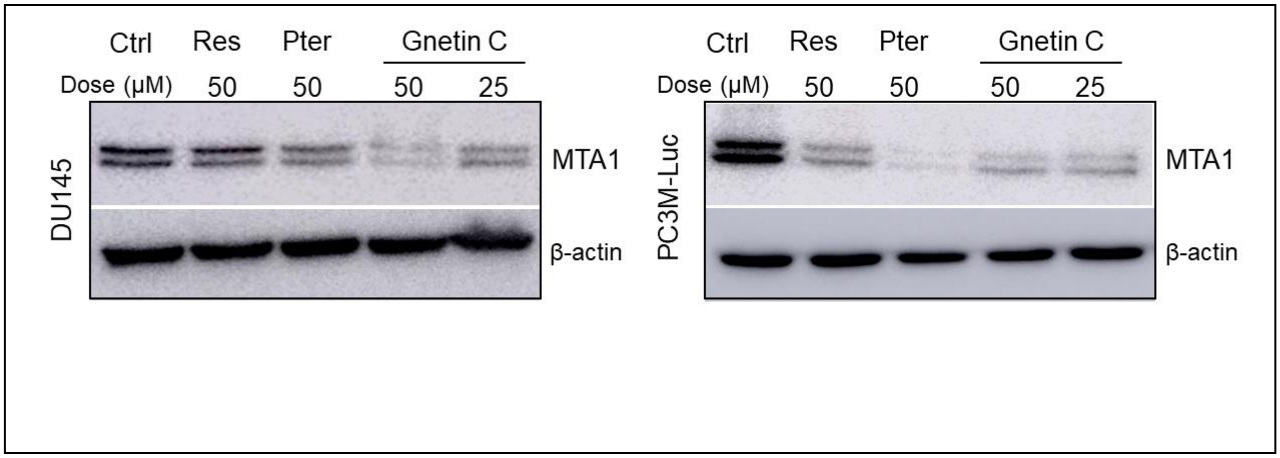

Figure S2. Stilbenoid compounds inhibit MTA1 expression in prostate cancer DU145 and PC3M-Luc cells. Gnetin C consistently demonstrated more potent inhibition of MTA1 compared to Res and Pter. Protein levels were assessed by western blot. β-actin was a loading control.

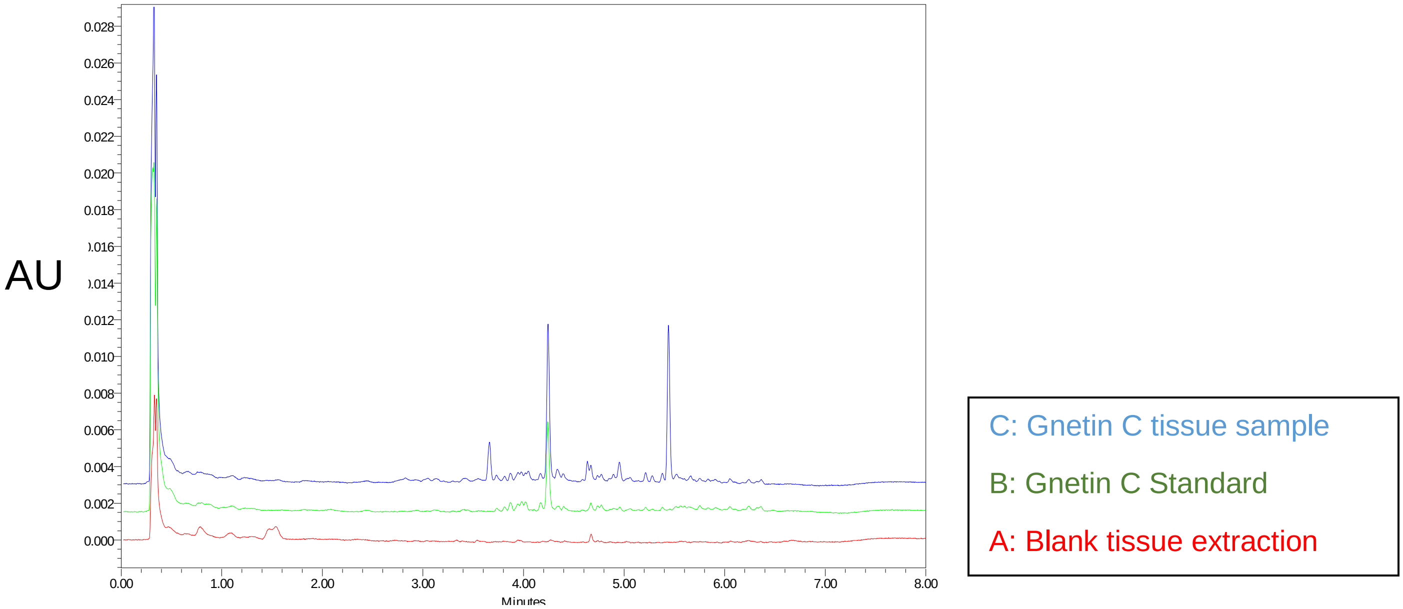

Figure S3. The overlaid UPLC chromatograms for A, blank tissue extraction; B, Gnetin C standard, 200 ng/ml; C, Gnetin C, 25 mg tissue sample

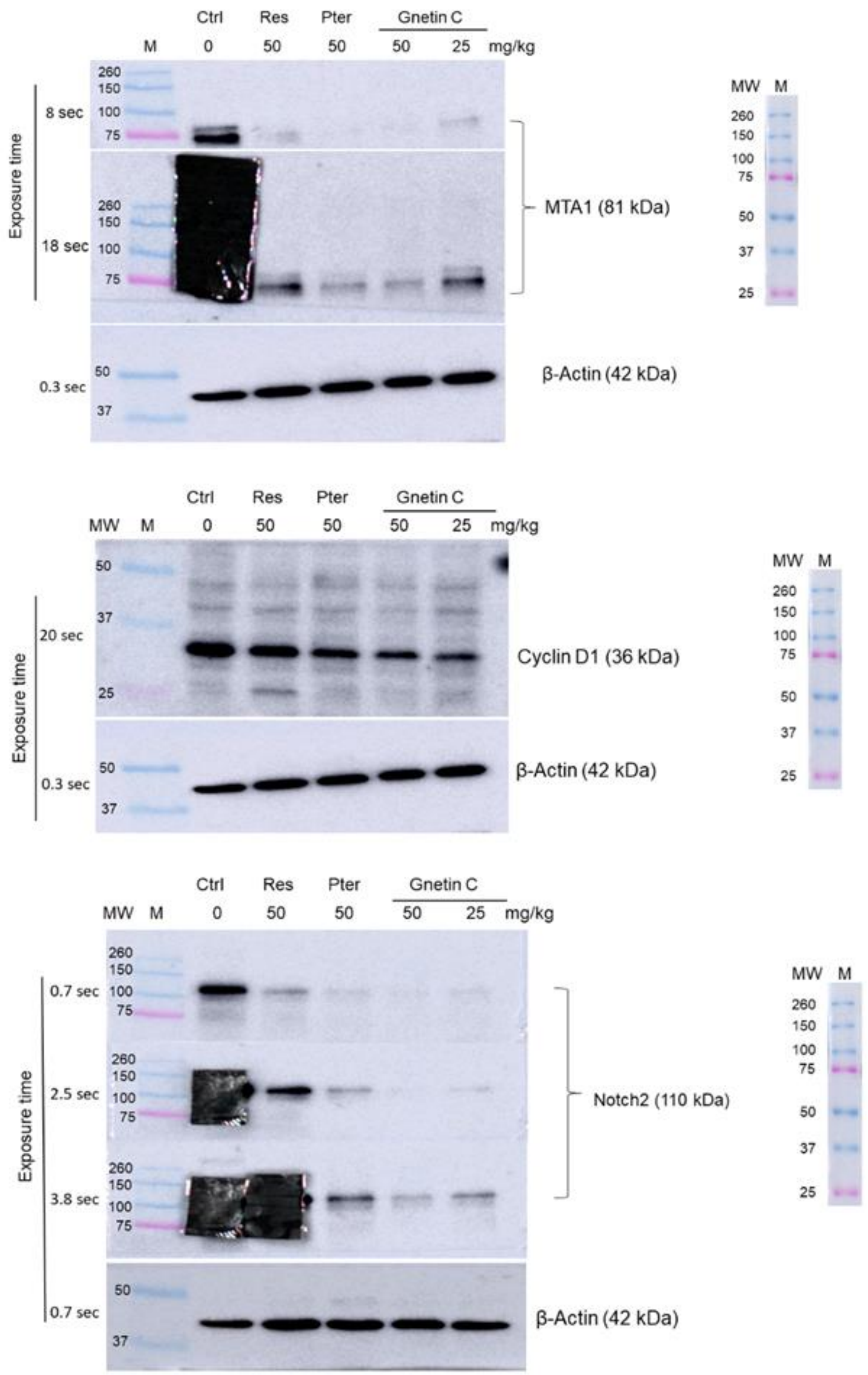

Figure S4. Original gels from Figure 6. Short exposure showed signals only for control tumors, in which expression of MTA1 (top gels) and Notch2 (bottom gels) were high. Masking control signals with foil and using longer exposure allowed us to detect lower expression of MTA1 and Notch2, and differences in tumors treated with compounds.

Table S1. Primary antibodies used in this study.

**Antibody**	**Method**	**Dilution**	**Source**	**Catalog #**
MTA1	Western Blot	1: 2500	Cell Signaling Technologies	5647
	IHC	1:50		
Cyclin D1	Western Blot	1:1000	Cell Signaling Technologies	2922S
Notch2	Western Blot	1:1000	Cell Signaling Technologies	57325
β-actin	Western Blot	1:2500	Santa Cruz Biotechnology	sc-69879
Ki67	IHC	1:50	Abcam	Ab16667
CC3	IHC	1:1000	Cell Signaling Technologies	9661S
CD31	IHC	1:500	Cell Signaling Technologies	77699
